# Pedunculate Oaks (*Quercus robur* L.) Differing in Vitality as Reservoirs for Fungal Biodiversity

**DOI:** 10.3389/fmicb.2018.01758

**Published:** 2018-08-03

**Authors:** Marta Agostinelli, Michelle Cleary, Juan A. Martín, Benedicte R. Albrectsen, Johanna Witzell

**Affiliations:** ^1^Southern Swedish Forest Research Centre, Swedish University of Agricultural Sciences, Alnarp, Sweden; ^2^Department of Natural Systems and Resources, School of Forest Engineers, Technical University of Madrid, Madrid, Spain; ^3^Department of Plant Physiology, Umeå Plant Science Centre, Umeå University, Umeå, Sweden

**Keywords:** fungal diversity, endophytes, tree vitality, *Quercus robur*, network analysis, phenolics

## Abstract

Ecological significance of trees growing in urban and peri-urban settings is likely to increase in future land-use regimes, calling for better understanding of their role as potential reservoirs or stepping stones for associated biodiversity. We studied the diversity of fungal endophytes in woody tissues of asymptomatic even aged pedunculate oak trees, growing as amenity trees in a peri-urban setting. The trees were classified into three groups according to their phenotypic vitality (high, medium, and low). Endophytes were cultured on potato dextrose media from surface sterilized twigs and DNA sequencing was performed to reveal the taxonomic identity of the morphotypes. In xylem tissues, the frequency and diversity of endophytes was highest in oak trees showing reduced vitality. This difference was not found for bark samples, in which the endophyte infections were more frequent and communities more diverse than in xylem. In general, most taxa were shared across the samples with few morphotypes being recovered in unique samples. Leaf phenolic profiles were found to accurately classify the trees according to their phenotypic vitality. Our results confirm that xylem is more selective substrate for endophytes than bark and that endophyte assemblages in xylem are correlated to the degree of host vitality. Thus, high vitality of trees may be associated with reduced habitat quality to wood-associated endophytes.

## Introduction

Trees growing in urban and peri-urban settings have high amenity value and support a variety of ecological and environmental benefits, including improvement of air quality and catchment of stormwater runoff (Brack, 2002; McPherson et al., 2011; Livesley et al., 2016). Urban and peri-urban trees may also act as important reservoirs, stepping stones or components of corridors for the biodiversity associated with trees (Bolund and Hunhammar, 1999; Dearborn and Kark, 2009). The ecological importance of these trees may be expected to increase even more in future, with shifts in land-use patterns and growing urbanization that promote fragmentation and patchiness of the landscapes (McKinney, 2002, 2006; Grimm et al., 2008; Dearborn and Kark, 2009). To guide the management of urban trees toward optimal procurement of multiple ecosystem benefits, more evidence-based information is needed about the different layers of their associated biodiversity.

A functionally significant part of tree-associated biodiversity is made up by the internal microfungi, called endophytes. These fungi colonize all tissues of trees, and generally remain asymptomatic. As a part of the “ecosystem microbiome” (Baldrian, 2016; van der Heijden and Hartmann, 2016) they contribute to important ecological processes, including microbial decomposition of organic material (Korkama-Rajala et al., 2008). Some tree-associated endophytes appear to influence the interactions of the tree host with pests and pathogens, either by shaping the tolerance and resistance responses of trees or directly influencing (i.e., antagonizing) the biotic intruders (DosSantos et al., 2015; Romeralo et al., 2015; Jia et al., 2016; Mefteh et al., 2017). In addition, some endophytes may shift to saprophytic or pathogenic life style under certain conditions (Müller et al., 2001; Schulz and Boyle, 2005; Saikkonen, 2007; Sieber, 2007; Porras-Alfaro and Bayman, 2011). Thus, fungal endophytes may act as an environment-specific, spatially and temporarily dynamic layer of biodiversity in trees, and as part of the hologenome they may influence the host trees’ phenotypic vitality (Albrectsen and Witzell, 2012; Witzell et al., 2014; Hardoim et al., 2015; Talbot, 2015).

Tree vitality is a phenotypic attribute that translates to the tree’s ability to grow and stay healthy in its prevailing environment (Shigo, 1991). Vitality can be evaluated using different morphological indicators, such as crown transparency, or metabolic traits (Chakraborty et al., 2013; Johnstone et al., 2013), such as tissue carbohydrate status (Wong et al., 2001; Dobbertin, 2005). With their ample carbohydrate resources, vital trees could be a favorable substrate for fungi. On the other hand, vital trees may possess well-functioning defensive mechanisms, allowing them to effectively resist native pathogen attacks (Wargo, 1996). Because even mutualistic fungi can initially be perceived as pathogenic invaders by their hosts (Zamioudis and Pieterse, 2012), trade-offs between a tree’s vitality and its value as a substrate for fungal biodiversity could occur: host defenses that effectively deter pathogens could also have negative effects on some beneficial symbiotic fungi (Hirsch and Mauchline, 2012). Martín et al. (2013) presented support for this view in a study on elm trees (*Ulmus* spp.) that were differentially susceptible to *Ophiostoma novo-ulmi*, a vascular pathogen causing Dutch elm disease (DED). They found less diverse and frequent infections by culturable endophytic fungi in xylem of elms with low susceptibility to DED, as compared to xylem of elms showing high DED susceptibility. Moreover, xylem phenolic composition differed between trees in different susceptibility classes, suggesting that defensive responses could explain the observed pattern in endophyte assemblages (Martín et al., 2013). It is, however, not known if this was a special response in elms, or if it is common that xylem-bound fungal communities are the more limited the more vital the host tree is.

The goal of this study was to add to the current understanding of the links between tree vitality and the diversity of endophytic fungal communities. We assumed that the more vital the trees, the stronger defensive mechanisms they exhibit, and that these mechanisms may limit infections by all fungi, including symbiotic endophytes. Accuracy of tree vitality was assessed via the analysis of phenolic metabolites in leaves. Phenolics are implicated as biomarkers for pathogen resistance in trees (Witzell and Martín, 2008; McPherson et al., 2014; Conrad et al., 2017), thus we used them to construct a proxy for the defensive status of each tree. Our hypothesis was that endophyte infections in perennial parts (bark and xylem) would be more limited in highly vital trees, as compared to trees with reduced vitality. Based on earlier studies (Martín et al., 2013), we expected this pattern to be stronger in xylem than in bark tissues. We tested our hypothesis by studying the culturable endophytes in bark and xylem of pedunculate oak (*Quercus robur* L.), a common tree species both in woodlands and green spaces, with relatively well characterized endophyte communities (Petrini and Fisher, 1990; Kehr and Wulf, 1993; Sieber et al., 1995; Ragazzi et al., 2003; Gonthier et al., 2006; Kwaśna et al., 2016). The results are discussed with emphasis on the importance of endophytes for tree health and biodiversity conservation in urban and peri-urban settings.

## Materials and Methods

### Oak Trees

Endophyte diversity was studied in 15–year-old *Quercus robur* trees with unknown genetic background. The site was originally planted in 2003 with 3-year-old seedlings at the edge of an agricultural field in Alnarp, on the outskirts of the city of Malmö in Southern Sweden (55°39′22″N, 13°05′35″E). The seedlings were planted in six groups of eight trees for a total of 48 trees at 4 m × 4 m spacing. Given the close proximity of trees to their neighbors, effects of surrounding vegetation, precipitation, temperature and soil conditions were expected to be rather similar, as well as their exposure to ambient inoculum.

### Assessment of Vitality

**FIGURE 1:**
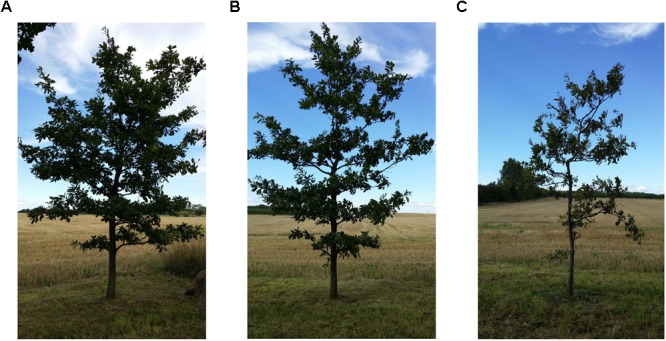
Even aged 15-year-old pedunculate oak trees planted in 2003 in Alnarp. In the picture the three vitality classes: high vitality **(A)**, medium vitality **(B)**, and low vitality **(C)**. Photo Marta Agostinelli.

Tree vitality was estimated on the basis of a visual assessment of the crown transparency (Müller and Stierlin, 1990) and the general condition of shoots, twigs, and leaves, and each tree was assigned to one of three vitality classes: high, medium, or low (**Figure 1**). Low vitality was not found to be associated with signs of specific pathogen or pest attacks. To investigate whether the phenotypic differences correlate with internal quality of the trees, we also analyzed phenolic compounds in oak leaves (where the metabolism of these compounds is active), as a proxy for the stress level and defensive capacity of the trees.

### Sample Collection

At the end of September 2014, eight trees representing each vitality class (according to **Figure 1**) were selected for sample collection (total *n* = 24 trees). From each tree, four asymptomatic twigs (approximately 30 cm in length) were collected between 1 and 2 m height, one from each of the four cardinal points. The twigs were transported in plastic bags to the laboratory where they were kept at 4°C degrees until further processing (within 24 h). From each twig, leaves were first removed and air-dried and then stored for analysis of phenolic compounds (see *analysis of phenolic compounds*) and a 5 cm long section was cut from the previous year’s growth. The section was surface sterilized using 70% ethanol and 4% sodium hypochlorite under aseptic conditions as described by Helander et al. (2007). Bark and xylem were separated, and four smaller pieces (approximately 3 mm × 3 mm × 1 mm) of each tissue type were cut from each twig and placed in one Petri dish (92 mm × 16 mm) containing 1.5% water agar. In total, 768 pieces were plated, i.e., 32 pieces per each tree: 16 pieces of xylem and 16 pieces of bark. The Petri dishes were incubated at room temperature in the dark for 4 weeks. During the incubation period, emerging colonies (569 in total) were recovered on 2% malt extract agar (MEA).

### Classification to Morphotypes

Based on visual examination, colonies were assigned to same morphotaxa (MT) if they shared several common macro-morphological characteristics: coloration, shape, elevation, edge, texture, presence or absence of extrolites on the surface, effect on medium, and growth rate. A colony was defined as a singleton MT if it could not be grouped with any other colony.

### DNA Extraction, Amplification, and Sequencing

Two representative specimens of each non-singleton MTs were selected for DNA extraction, with the exception of those that had only one or no viable specimen left (MT 21 and MT28, respectively), and those that were identified from a previous study (Martín et al., 2015) (MT 1). In addition, three MTs were morphologically matched to another endophyte collection and representative samples were then selected for DNA analyses (MT 3, MT 9, and MT 14) (**Table 1**). Plugs of pure cultures growing on MEA were transferred to Petri dishes containing malt extract broth and incubated for 2–4 weeks at room temperature in the dark. Mycelia were then filtered, placed in 50 mL Falcon tubes and lyophilized for 48 h. Samples were pulverized in a FastPrep^®^-24 homogenizer (MP Biomedicals, Santa Ana, CA, United States). Genomic DNA was extracted with E.Z.N.A. SP Plant DNA kit (Omega Bio-Tek, Inc., Norcross, GA, United States) following manufacturer’s instructions. DNA quantity and quality was measured using a NanoDrop^®^ ND-1000 spectrophotometer (Wilmington, DE, United States). The internal transcribed (ITS) region of rDNA was amplified by PCR using general primers ITS1 and ITS4 (White et al., 1990). The PCR was performed in 50 μL reactions and consisted of: 1 × PCR Buffer, 200 μM of dNTPs, 750 μM of MgCl_2_, 0.01 μM DreamTaq polymerase (5 U/μL), 0.4 μM of each primer, and 10 ng μL^-1^ of DNA. The PCR program started with denaturation at 95°C for 5 min, followed by 33 cycles of 95°C for 30 s, annealing at 57°C for 30 s, and 72°C for 30 s, followed by a final extension step at 72°C for 7 min. To check if PCR was successful, the PCR products were visualized under UV light by gel electrophoresis on 1.5% agarose gel dyed with GelRed^TM^. PCR products were purified with Agencourt AMPure XP (Agencourt Bioscience Corp, Beverly, MA, United States) following manufacturer’s instructions. After quantification of amplified products with Qubit fluorometer 3.0 (Life Technologies, Sweden), samples were sent for Sanger sequencing at National Genomics Infrastructure (NGI) at Science for Life Laboratory (SciLifeLab, Uppsala, Sweden).

**Table 1:** List of putative taxa of morphotypes (MT 1-30) isolated from pedunculate oak (*Quercus robur* L.) twigs and their relative abundance (no. emerged isolates belonging to MT pooled per twig/total no. of pooled isolates recovered) in different vitality classes (L, low; M, medium; and H, high) and in different tissue types (B, bark; X, xylem).

MT No.	Putative taxon	Isolates	Relative abundance by vitality and tissue					Genbank accession numbers
			H	M	L	B	X	
1	*Aureobasidium pullulans*	5	0.3	0.3	1.0	0.3	1.3	JX462673.1^† ∗∗∗^
2	*Melanconiella chrysomelanconium*	2	0.3	0.3	0	0.7	0	KX815458; KX815459
3	*Tubakia dryina*	43	4.9	3.9	5.2	12.7	1.3	KX815460^†^; KX815461^†^
4	Unknown	7	1.0	0.3	1.0	2.0	0.3	^∗^
5	*Lophiostoma corticola*	11	0.7	1.0	2.0	3.3	0.3	KX815462; KX815463
6, 14	*Pyrenochaeta cava*	2	0.3	0.3	0	0.7	0	KX815464; KX815465 KX815477^†^; KX815478^†^
7	*Pezicula sporulosa*	2	0.7	0	0	0.7	0	KX815466; KX815467
8	Unknown	3	0	0	1.0	1.0	0	^∗^
9	*Phoma sp. 1*	5	0.7	0	1.0	1.6	0	KX815468^†^; KX815469^†^
10	*Dothideomycetes sp. 1*	11	0.3	1.6	1.6	3.6	0	KX815470; KX815471
11	*Sordariomycetes sp. 1*	12	1.6	0.7	1.6	3.9	0	KX815472; KX815473
12	*Amphiporthe leiphaemia*	3	0	0.3	0.7	1.0	0	KX815474; KX815475
13	*Pezicula sp.1*	18	1.3	0.7	3.9	2.0	3.9	KX815475; KX815476
15	*Phaeomoniella sp. 1*	10	1.0	0.7	1.6	2.3	1.0	KX815479; KX815480
16	*Sordariomycetes sp. 2*	4	0	0.7	0.7	1.3	0	KX815481; KX815482
17	Unknown	2	0.3	0	0.3	0.3	0.3	^∗^
18	*Pleosporineae sp. 1*	5	0.7	0.7	0.3	0.7	1.0	KX815483; KX815484
19	*Dothideomycetes sp. 2*	3	0.3	0	0.7	0.3	0.7	KX815485; KX815486
20	*Ascomycetes sp.1*	4	0	0	1.3	0	1.3	KX815487; KX815488
21	*Phoma sp. 2*	2	0	0	0.7	0	0.7	KX815489;
22, 23	*Colpoma quercinum*	57	6.9	6.9	4.9	17.6	1.0	KX815490; KX815491; KX815492
24	Unknown	3	0.7	0.3	0	0.7	0.3	^∗^
25	Unknown	3	0	0.7	0.3	0.3	0.7	^∗^
26	Unknown	2	0.3	0	0.3	0.7	0	^∗^
27	*Ascomycetes sp.2*	11	1.3	2.0	0.3	3.6	0	KX815493; KX815494
28	Unknown	7	0.7	1.3	0.3	2.0	0.3	^∗^
29	*Eurotiomycetes sp. 1*	15	1.3	1.0	2.6	4.6	0.3	KX815495; KX815496
30	Unknown	54	3.6	6.2	7.8	11.1	6.5	^∗∗^

*GenBank accession numbers indicate the GenBank identifier of the corresponding ITS sequences. ^†^Specimen morphologically matched to previous culture collection. ^∗^ Specimen not sequenced. ^∗∗^ Combined group of singletons. ^∗∗∗^ Endophyte identified in previous study.*

### Sequence Analysis

The raw sequence data were visualized with Chromas (software version 2.4.4, Technelysium, South Brisbane, Australia). Among the samples sequenced, one was excluded from further analyses because of contamination. All sequences were aligned and edited with SeqMan Pro^TM^ (©1988–2016, DNASTAR, Inc., Madison, WI, United States) software. Putative taxa were assigned by comparing the acquired sequences to those in the reference database at NCBI^1^ through BLASTN search. The ITS sequence homology for delimiting fungal taxa was set to >98.5% for presumed fungal species) and 94–97% for determination at the genus level. ITS sequence information was deposited in GenBank (NCBI) with accession numbers KX815458-KX815496 (**Table 1**).

### Analysis of Phenolic Compounds

To gain information about the defensive status of the trees, leaf material that was removed from the twigs was air-dried for analysis of phenolics compounds. Four healthy-looking leaves from each tree were pooled to one sample (*n* = 24) and each sample was milled into a homogenous powder using an MM301 ball mill (Retsch GmbH). The extraction and HPLC analysis was done following the procedure described in Romeralo et al. (2016). From each sample, a subsample of 10 mg, together with three glass pearls and 1 mL cold methanol were added in an Eppendorf vial. The samples were shaken in the mill for 2 min, centrifuged for 2 min at 18 928 *g*, and the clear supernatant was transferred into another vial. The procedure was repeated with another 500 μL solvent and the supernatants were combined. The solvent was evaporated to dryness in a vacuum concentrator and the extract was stored at 4°C until analysis (within 1–3 days from extraction). Before the analysis, the extracts were dissolved in 400 μL methanol:water (1:1, v:v) and passed through disposable filters (0.45 μ pore size) to remove any particles. The HPLC system consisted of a Merck Hitachi LaChrom device with a D-7100 pump, D-7200 autosampler, D-7300 column oven at 40°C, and a D-7455 DAD detector scanning the absorbance between 220 and 400 nm. Injection volume was 20 μL and separation was achieved on a 100 mm HyPurity C18 (Thermo Scientific) column using a gradient made of water (acidified with o-phosphoric acid to pH 3; A) and methanol (B) as follows: 10% B (0–1 min); 10–70% B (1–20 min); 70% B (20– 23 min); 70–100% B (23–30 min); followed by flushing and equilibration to initial conditions (flow rate 0.8 mL min^-1^). The peak area (AU) was normalized toward dry weight of each sample (mg).

### Data Compilation and Analyses

Colonization frequency was calculated as the proportion (%) of bark and xylem samples with at least one emerging colony. To estimate endophyte diversity, MT richness (S^MT^) (Whittaker, 1972) was calculated as the number of different MTs recovered per twig. To assess the commonness/rarity of an individual MT, its relative abundance was calculated as the sum of isolates belonging to that MT, divided by the total number of isolates emerged from all samples in that tissue type.

Because of the non-normal distribution of the data, Mann–Whitney *U* (W) test was used to test the difference in CF and S^MT^ values between bark and xylem. Kruskal–Wallis (KW) test was used to determine differences in CF and S^MT^ values among the orientation and the vitality classes (Castillo Lopez et al., 2014). Dunn’s *post hoc* test was applied if there was significance difference. R statistical software was used in all analysis unless otherwise specified (R Core Team, 2013).

To compare taxa richness in bark and xylem of trees in different vitality classes, individual-based rarefaction curves were constructed with EstimateS 9.1.0 (Colwell et al., 2004) using 100 randomizations, sampling without replacement and using default settings for upper incidence limit for infrequent species.

To assess the similarity of fungal communities in different tissues and vitality classes, Jaccard index was calculated based on the presence-absence of the taxa (J_p-a_, *vegdist* function, vegan package, Oksanen et al., 2016). In addition, Chao-Jaccard index was calculated based on species abundance including rare unseen shared species (J_ab_, *dis.chao* function, CommEcol package) (Jost et al., 2011). Venn diagrams were used to visualize the core community of oak endophytes (Shade and Handelsman, 2012). The variation in shared MTs between tissue types and vitality classes was visualized considering species’ presence-absence and species similar abundance (Shade and Handelsman, 2012). Variability in the endophyte communities in different tissues and vitality classes was tested on pooled abundance data with permutational multivariate analysis of variance (PERMANOVA, *adonis* function) based on Bray-Curtis dissimilarity matrix (vegan package). Homogeneity of variance among groups was tested using permutational test of multivariate group dispersions implemented as *betadisper* in the vegan package (Oksanen et al., 2016).

Nestedness (N) analysis (R package bipartite) was used to measure the structure of the morphotype system in the tissue and vitality class of pedunculate oak. The associations among morphotypes, vitality classes and tissue types were then visualized with bipartite network graphs (R package bipartite). Logistic regression analysis was then performed to predict the probability of finding a given morphotype based on tissue type, vitality class, and tissue–vitality interaction (JMP^®^ Pro 13, SAS Institute Inc., Cary, NC, United States).

To examine differences in phenolic profiles between the trees classified to different vitality classes, the peak area values (AU/mg) of a total of 10 UV-absorbing peaks and vitality class were used as input variables in quadratic canonical discriminant analysis (JMP^®^ Pro 13, SAS Institute Inc., Cary, NC, United States).

## Results

A total of 569 fungal isolates were recovered from 768 bark and xylem samples of pedunculate oak. The emerged colonies were initially grouped into 29 MTs and one mixed group of unidentified singletons [(MT 30) which comprised 54 specimens in total]. After DNA analysis, the number of MTs was reduced to 27 since sequence similarity putatively indicated the same species for MT 6 and MT 14 as well as MT 22 and MT 23 (**Table 1**). Seven MTs were not sequenced because there were no viable cultures or they did not grow on malt extract broth. All remaining MTs belonged to Ascomycota that were putatively assigned to *Dothidiomycetes, Sordariomycetes, Leotiomycetes, and Eurotiomycetes* and two unassigned Ascomycota classes. Of those, eight were putatively identified to species level and four to genus level. The singletons (MT30) were not sequenced (**Table 1**).

### Colonization Frequency (CF)

**FIGURE 2:**
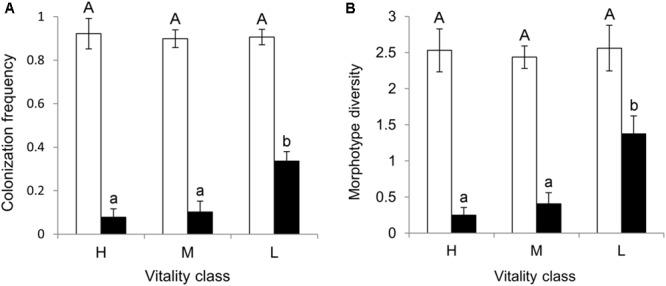
**(A)** Colonization frequency (CF = proportion of bark and xylem samples with at least one emerging colony) and **(B)** morphotype diversity (S^MT^ = number of different MTs recovered per twig) mean values in bark (white bars) and xylem (black bars) of pedunculate oak twigs (*n* = 32) collected from trees (*n* = 8) in three vitality classes (H, high; M, medium; L, low). Shown are the mean values of eight replicate trees (±SE) with different letters indicating significant difference among vitality classes (Kruskal Wallis test, *p* < 0.05 in xylem tissues for both CF and S^MT^).

Bark tissue yielded more endophyte isolates than xylem (484 isolates and CF = 91% in bark, 85 isolates and CF = 17% in xylem; Mann–Whitney *U* = 574.5, *n*_1_ = *n*_2_ = 2, *p* < 0.0001). The CF of bark samples did not differ among the three vitality classes (χ^2^ = 0.67, *p* = 0.71; **Figure 2A**). In contrast, the CF of xylem samples differed among the vitality classes, being highest in low vitality trees (Kruskal–Wallis χ^2^ = 16.28, *p* = 0.0003; **Figure 2A**). Dunn’s *post hoc* test results showed a significant difference in xylem CF between low and high vitality class (*p* = 0.0001) and low and medium vitality class (*p* = 0.0004). No significant difference was found between high and medium class (*p* = 0.38). Sample orientation (N, S, W, E) and tree (individual) had no significant effect on CF (Kruskal–Wallis χ^2^ = 2.84, *p* = 0.41 and Kruskal–Wallis χ^2^ = 24.68, *p* = 0.36, respectively) and were not considered as factors in further analyses.

### Morphotype Richness (S^MT^)

**FIGURE 3:**
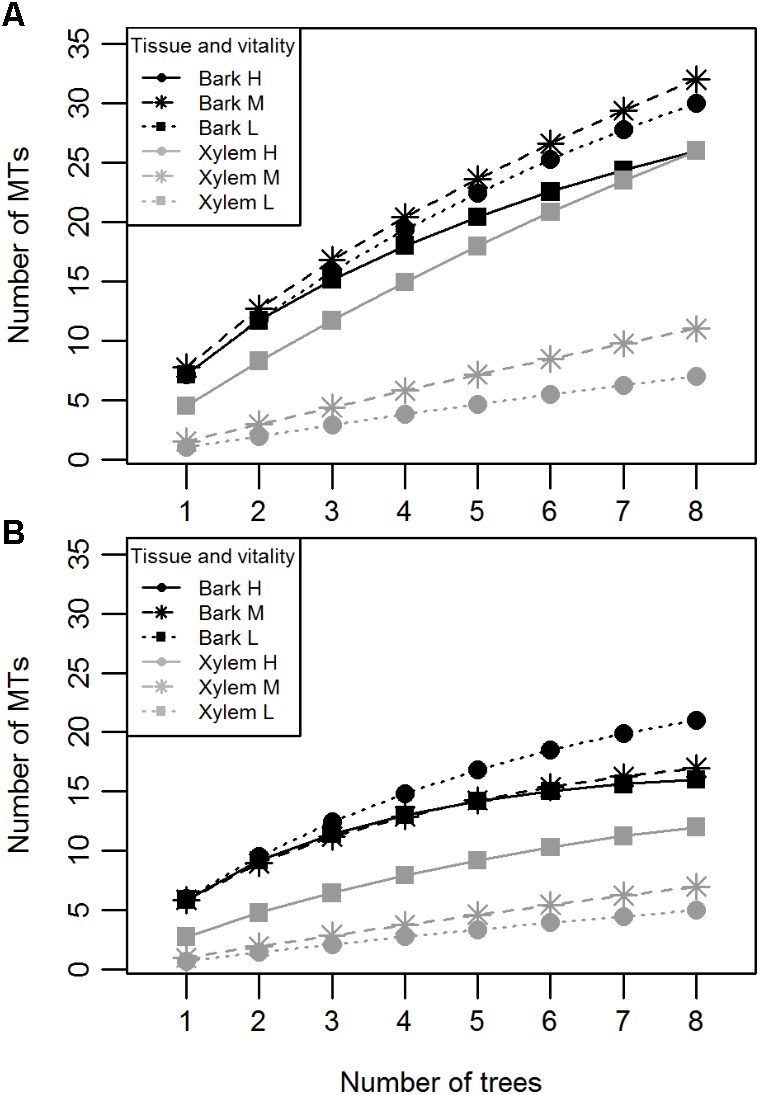
Individual-based rarefaction curves of pedunculate oak endophytes indicating the number of MTs isolated per tissue (bark or xylem) and vitality class (H, high; M, medium; L, low) with **(A)** and without **(B)** singletons.

Bark and xylem differed in the number of different MTs they hosted (Mann-Whitney *U* = 552.0, *n*_1_ = *n*_2_ = 2, *p* < 0.000); 79% of the MTs were found in bark, whereas 21% were found in xylem (**Table 1**). Similarly to CF, S^MT^ of bark samples did not differ among the vitality classes (Kruskal–Wallis χ^2^ = 0.49, *p* = 0.78; **Figure 2B**), whereas that of xylem samples did; low vitality samples yielded a higher number of MTs than medium and high vitality samples (Kruskal–Wallis χ^2^ = 16.58, *p* = 0.0002; **Figure 2B**). Dunn’s *post hoc* test showed a significant difference between low and high vitality class (*p* = 0.0001) and low and medium vitality class (*p* = 0.0005). No significant difference was found between high and medium class (*p* = 0.34). Sample orientation (N, S, W, E) and tree (individual) had no significant effect on S (Kruskal–Wallis χ^2^ = 0.11, *p* = 0.99 and Kruskal–Wallis χ^2^ = 24.67, *p* = 0.36, respectively) and were not considered as factors in further analyses. Individual-based rarefaction curves constructed for tissue type and vitality class confirmed these results for tissue type and vitality class. Only the curve of low vitality bark samples approached the asymptote (**Figure 3A**). When the singletons were removed from the analysis, the shape of all curves improved (**Figure 3B**).

### Fungal Community Diversity of Tissues and Vitality Classes

**FIGURE 4:**
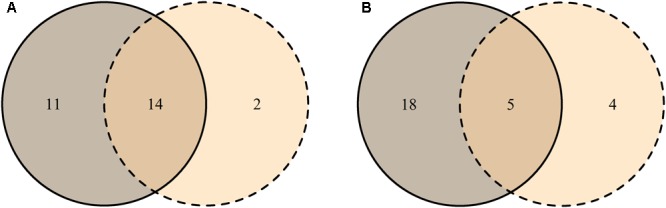
Venn diagrams representing the distribution of unique MTs in bark (solid line) and xylem (dashed line), and MTs shared between the two tissues (overlapping area) based on the presence-absence **(A)** and on the similar abundance in the tissue **(B)**. The numbers indicate how many morphotypes were found in each tissue and shared between the tissues. MT30 is not included since it represents the group of singletons.

**FIGURE 5:**
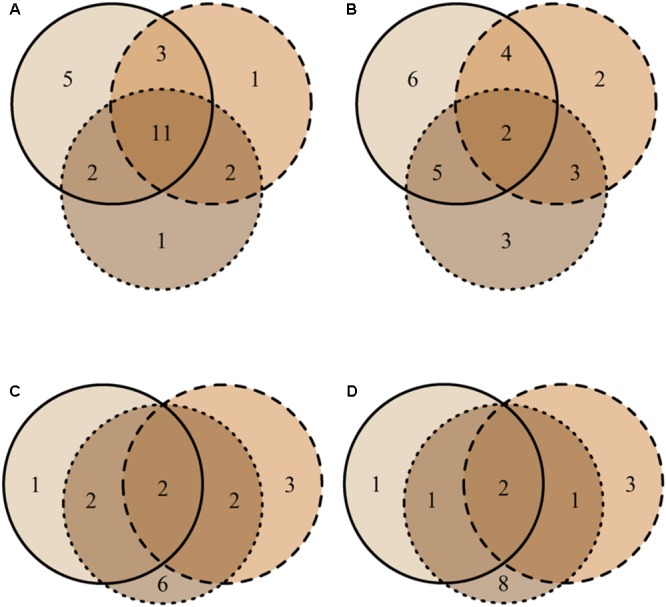
Venn diagrams representing the distribution of unique MTs found in high vitality class (solid line), medium class (dashed line), low vitality class (dotted line), and MTs shared among the three vitality classes (overlapping areas) based on their presence-absence **(A)** and similar abundance **(B)** in bark and the presence-absence **(C)** and similar abundance **(D)** in xylem. The numbers indicate how many morphotypes were found in each vitality class and shared among vitality classes. MT30 is not included since it represents the group of singletons.

Most MTs were found in both tissue types (J_p-a_ = 0.82), though their abundance differed (J_ab_ = 0.34) (**Figures 4A,B**). Based on PERMANOVA (abundance), bark and xylem fungal communities differed significantly (*R*^2^ = 0.17, *p* = 0.001). Most MTs recovered in bark were found in all three vitality classes, resulting in similar fungal compositions (J_p-a_L-M = 0.71, J_p-a_L-H = 0.70, J_p-a_M-H = 0.71). However, according to the MTs similar abundance the differences among classes increased (J_ab_L-M = 0.34, J_ab_L-H = 0.31, J_ab_M-H = 0.32) (**Figures 5A,B**), even though PERMANOVA (abundance) analysis did not provide statistically significant differences (*R*^2^ = 0.07, *p* = 0.71). Most MTs were found in low vitality xylem, however, the xylem communities of the three vitality classes resulted to be rather similar considering the MTs presence/absence (J_p-a_L-M = 0.88, J_p-a_L-H = 0.86, J_p-a_M-H = 0.87) and MTs similar abundances (J_ab_L-M = 0.71, J_ab_L-H = 0.70, J_ab_M-H = 0.71) (**Figures 5C,D**). Based on PERMANOVA (abundance), xylem-associated fungal communities did not differ between high and low vitality classes (*R*^2^ = 0.06, *p* = 0.73) nor between high and medium vitality class (*R*^2^ = 0.16, *p* = 0.37). Instead, there was a difference in fungal community composition between the medium and low vitality class (*R*^2^ = 0.15, *p* = 0.03).

### Nestedness Analysis and Logistic Regression

**FIGURE 6:**
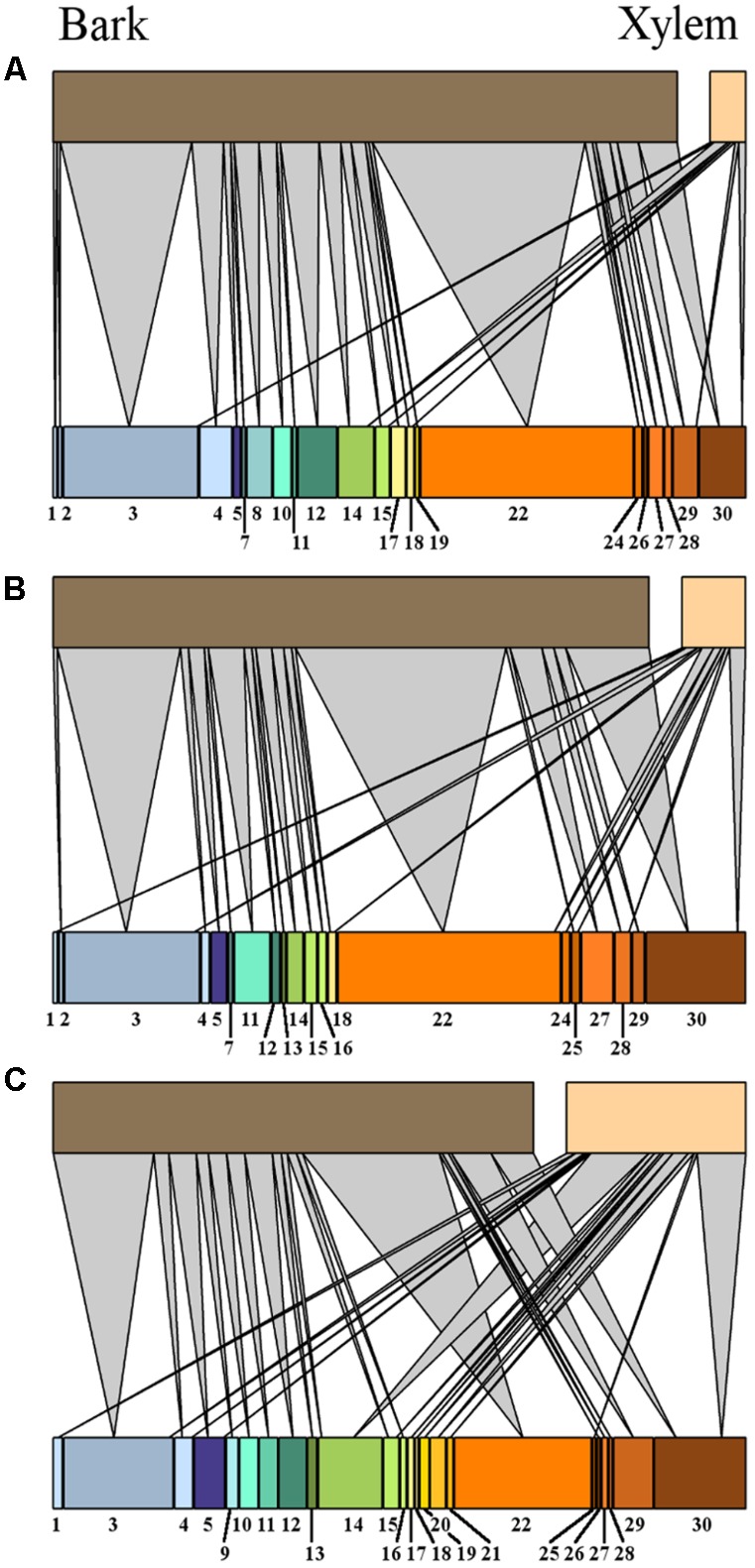
Bipartite network graphs representing the isolates relative abundance of fungal isolates, arranged after morphotype (indicated by number) in oak tissues (xylem and bark) from high **(A)**, medium **(B)**, and low **(C)** tree vitality classes.

The MTs’ associations with tissue types and vitality classes were visualized using bipartite graphs (**Figure 6**). The web structure between tissue and vitality class was more complex in xylem of low vitality trees (*N* = 37.2) than in medium (*N* = 15.9), or high (and *N* = 4.5) vitality trees (**Figure 6**). The logistic regression showed that tree vitality (*P* > χ^2^ = 0.0002), tissue type (*P* > χ^2^ = 0.0001), and the interaction between vitality and tissue type (*P* > χ^2^ = 0.0029) influenced the endophytic community composition (**Table 2**). The presence of *Pyrenochaeta cava* (MT14) was positively correlated to low vitality class. The presence of some MTs was positively correlated with tissue type (*Tubakia dryina* and MT4 in bark) or with the combination of tissue type and vitality class (i.e., MT8 in high vitality bark) (**Table 2**).

**Table 2:** Logistic regression analyses were performed to explore the data for morphotypes that divided the data set according to vitality (on the left) and tissue type (on the right).

Morphotype		Vitality, *N* = 768, DF = 2			Tissue type, *N* = 768, DF = 1		
		-LogLikelihood	ChiSq	*P* > ChiSq	-LogLikelihood	ChiSq	*P* > ChiSq
MT1		0.75	1.49	0.47	0.97	1.94	0.16
MT2		0.81	1.62	0.44	1.39	2.78	0.10
MT3	^∗∗^	0.13	0.26	0.88	55.66	111.31	0.0000
MT4	^∗∗^	2.47	4.95	0.08	8.13	16.26	0.00000
MT5	^∗∗^	3.24	6.49	0.039	7.48	14.96	0.0000
MT7		0.81	1.62	0.44	1.39	2.78	0.10
MT8	^∗∗∗^	7.75	15.51	0.0004	4.88	9.77	0.0018
MT9	^∗∗∗^	4.42	8.83	0.012	2.78	5.57	0.018
MT10	^∗∗∗^	4.55	9.09	0.011	7.70	15.41	0.0000
MT11	^∗∗∗^	3.81	7.61	0.022	11.26	22.52	0.0000
MT12	^∗∗∗^	3.79	7.59	0.023	15.57	31.15	0.0000
MT13	^∗∗^	2.15	4.31	0.116	2.78	5.57	0.018
MT14	^∗^	6.83	13.65	0.0011	1.84	3.68	0.055
MT15		0.26	0.51	0.77	1.59	3.19	0.07
MT16	^∗∗^	1.63	3.25	0.20	2.78	5.57	0.018
MT17		2.79	5.58	0.06	0.34	0.69	0.41
MT18		0.22	0.44	0.80	0.10	0.20	0.65
MT19		2.15	4.31	0.12	0.53	1.05	0.31
MT20	^∗∗∗^	5.53	11.05	0.0040	3.48	6.96	0.0083
MT21		2.20	4.41	0.111	1.39	2.78	0.10
MT22	^∗∗^	1.45	2.90	0.24	116.69	233.38	0.0000
MT24		1.63	3.25	0.20	0.00	0.00	1.00
MT25		1.39	2.78	0.25	0.17	0.34	0.56
MT26		0.81	1.62	0.44	1.39	2.78	0.10
MT27	^∗∗^	2.04	4.08	0.13	9.83	19.67	0.0000
MT28	^∗∗^	1.01	2.02	0.36	2.00	4.00	0.046
MT29	^∗∗∗^	3.52	7.03	0.030	12.12	24.25	0.0000
MT30	^∗∗∗^	3.20	6.40	0.041	4.62	9.24	0.0024

*^∗^MTs whose presence was affected by vitality, ^∗∗^MTs whose presence was affected by tissue type, and ^∗∗∗^MTs whose presence was affected by tissue type–vitality interaction.*

### Leaf Phenolic Composition in Vitality Classes

**FIGURE 7:**
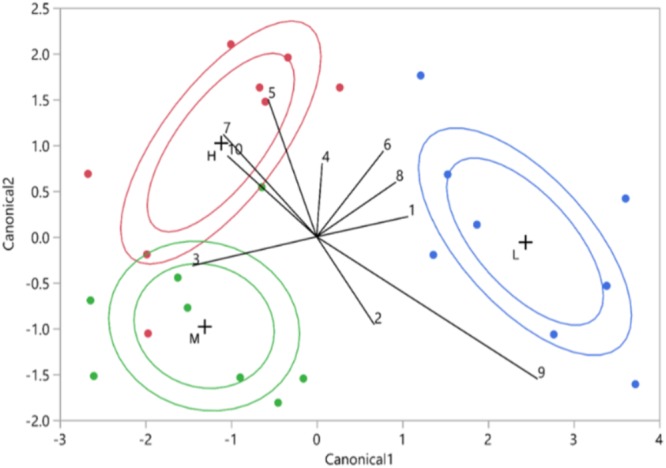
Vitality class based on canonical discriminant analysis of HPLC data (10 UV-absorbing peaks. The red points represent the high vitality leaves (H), the green points the medium vitality leaves (M), and the blue points the low vitality leaves (L). The internal ellipsoid line represents the normal ellipse region estimated to contain the 50% of the population of the group. The external ellipsoid line represents the confidence region containing the true mean of the group with 95% accuracy. The numbers represent the phenolics from the 10 biggest phenolic peaks.

**FIGURE 8:**
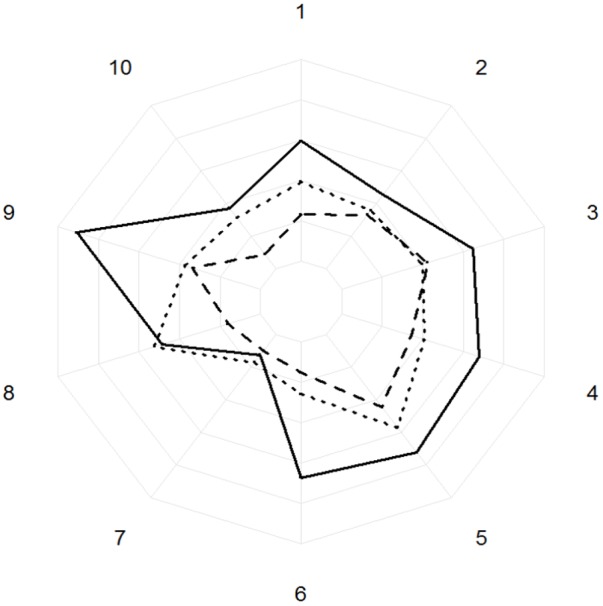
Areas under the curve of the peaks of absorbance (AU/mg) of the ten biggest phenolics peaks for high (dotted line), medium (dashed line), and low (solid line) vitality classes. The concentric circles represent the areas under the curve with an increasing of 10,000 AU/mg at every concentric circle. The numbers represent the phenolics from the 10 biggest phenolic peaks.

The phenolic compounds detected in oak leaves were putatively identified as quercetin derivatives (compound no. 1, 2, 3, 5, 6, 8, and 10; **Figures 7, 8**) and kaempferol derivatives (compound no. 7 and 9; **Figures 7, 8**). One compound (no. 4; **Figures 7, 8**) remained unclassified (λmax at 251 nm and a distinctive additional shoulder at 351 nm). The canonical discriminant analysis of oak leaves showed a clear division among the phenolic profiles of the leaves of the vitality classes with each class separating from the other two (**Figure 7**). Among the ten dominating phenolics’ peaks, highest levels were detected in low vitality trees, while lowest levels were observed in medium vitality trees (**Figure 8**).

## Discussion

Our study provided support for the hypothesis that fungal endophyte communities are limited in highly vital trees. We found that xylem infections by culturable endophytes were more frequent in low vitality trees than in high or medium vitality trees. In addition, the taxonomic richness was lowest in the xylem of high vitality oaks. Similar results were found in the roots of oaks stressed by floods (Kwaśna et al., 2016). Venn diagrams and similarity indices revealed that while most MTs were shared in both tissues and all vitality classes, their abundance could differ substantially. Furthermore, we found highest nestedness among MTs in xylem of low vitality trees, possibly reflecting the turnover of taxa as tree decline progresses but also higher interaction among taxa with similar functional guild (Rezende et al., 2007). In some studies, the level of nestedness has been suggested to correlate positively with commensalistic networks, i.e., reduced competition and increased robustness of interaction networks (Piazzon et al., 2011), while it correlates negatively with mutualistic and antagonistic networks (Blüthgen et al., 2008; Piazzon et al., 2011). Thus, our results indicate that the endophytic populations of high and medium vitality trees are formed mainly by mutualistic or antagonistic groups of endophytes whose interactions happen more within groups than among groups of endophytic taxa. On the other hand, the higher nestedness found in low vitality trees suggests that low vitality trees’ endophytic population might have shifted toward a more commensalistic lifestyle that increased the interactions among groups of endophytic taxa. We also found that connectivity, which indicates the proportion of possible links actually observed in a web (Blüthgen et al., 2008) was highest in the intermediate vitality trees, suggesting that the endophytic community of intermediate trees varies slightly less than high and low vitality trees’ endophytic community. In general, our findings suggest that the fungal communities in highly vital trees may have been biased toward antagonistic interactions, which could explain the low number of infections and MTs found in the xylem. Yet, endophyte communities in these trees might also be impoverished in terms of the architecture of their interactions. Endophytes tend to assume a quiescent (latent) stage following penetration of healthy tissues (Sieber, 2007). Thus, the fungal–fungal interactions might be less active in highly vital tissues than in tissue expressing low vitality.

The taxonomic diversity detected in our study resembled that described in previous studies on oak endophytic communities (Petrini and Fisher, 1990; Kowalski and Kehr, 1992; Ragazzi et al., 2003). The MTs of our study belong mainly to the classes *Leotiomycetes, Eurotiomycetes, Sordariomycetes*, and *Dothideomycetes* that are reported as common classes among tree endophytes in previous studies (Arnold et al., 2007; Higgins et al., 2007; Rodriguez et al., 2009). We recovered generalist endophytes found in other tree species and also endophytes specific to oak. For instance, *Amphiporthe leiphaemia* is an endophyte specific to pedunculate oak while *Colpoma quercinum* has been recovered in several oak species in Europe and Asia (Ragazzi et al., 2003; Sieber, 2007; Sun et al., 2012). *Tubakia dryina, Pezicula spp., P. cava, Phoma spp., Lophiostoma corticola*, and *Aureobasidium pullulans* are generalist species found in different hosts. Other common and generalist species found in previous studies of oak twigs, i.e., *Alternaria alternata, Phomopsis sp., Trichoderma sp.* (Ragazzi et al., 2003; Gonthier et al., 2006; Sieber, 2007; Sun et al., 2012) were not recovered in our study. *Melanconiella chrysomelanconium* was infrequently isolated in our sampling (abundance <0.7%, **Table 1**). Interestingly, this species was recently described by Voglmayr et al. (2012) as highly host-specific to *Carpinus betulus*, which was not found in the immediate vicinity of the oaks we studied. More research would be needed to clarify the frequency of this fungus on oaks.

Because trees are long-living organisms and are subjected to multiple endophytic infections along their life span, it has been difficult to address how endophytes might influence tree characteristics, i.e., the phenotypic vitality. Moreover, it has also been difficult to understand the functions of endophytes, most of which are still unknown. Some of the identified morphotypes may contribute to the health of the oaks as antagonist to pathogenic fungi (Schulz et al., 1995; Sullivan and White, 2000; Mousa and Raizada, 2013; Martín et al., 2015). For instance, *P. cava* has been found to be primary associated with elms resistant to *Ophiostoma novo-ulmi* (Martín et al., 2013). Among the unidentified morphotypes, MT8, having being found only in high vitality trees, might be considered a vigor biomarker for oak; albeit its identity still remains unknown. Further research would be interesting to validate this association. On the other hand, some other fungi recovered in our study, such as *A. leiphaemia, C. quercinus, T. dryina, Pezicula spp.*, and *Phoma spp.* have been reported in the literature as latent pathogens or saprotrophs (Schulz et al., 1995; Sieber et al., 1995; Ragazzi et al., 2003; Gonthier et al., 2006; Kowalski, 2006; Sieber, 2007; Aveskamp et al., 2010; Sun et al., 2012; Harrington and McNew, 2017). *Pezicula* species, despite being possible latent pathogens, can produce secondary metabolites with anti-fungal properties (Mousa and Raizada, 2013). The two *Pezicula s*pecies recovered in our study caused discolouration of the MEA suggesting that these species might be actively producing extracellular secondary metabolites in their environment. Despite some endophytes are known to positively affect their host, it is also known that fungal endophytes are ubiquitous fungi whose lifestyle might change under certain circumstances (Rodriguez and Redman, 2008; Rodriguez et al., 2009; Rai and Agarkar, 2014). The function and lifestyle of fungal endophytes might be dynamic and undergo changes due to their host’s biochemical and genetic responses to the infections and to the dynamics created by the fungal–fungal interactions in the plants (Rodriguez et al., 2004). It is thus challenging to determine the precise roles of endophytic species in their hosts. For this purpose, a recent study by Blumenstein et al. (2015b) showed that Phenotype MicroArray is a valuable tool to investigate endophytes phenotypes and their substrate preferences, particularly the response of endophytes to specific chemical compounds and their reaction to substrate competition (Blumenstein et al., 2015a). This method combined with network analysis can help improve our understanding of endophytes complex interactions with their hosts (David et al., 2016).

Because all trees sampled in our study were planted at the same time in the same, limited area, we can assume that all trees were exposed to a similar inoculum pressure of horizontally spreading endophytes (Rodriguez et al., 2009). Thus, we expect that the internal tree quality associated to the vitality status and tissue type may have shaped the endophyte communities. To study the internal quality of trees, we constructed a proxy for the defensive status of each tree, based on levels of phenolic metabolites in leaves, where the most active biosynthesis of these compounds is likely to take place. We focused on phenolics because these compounds have been implicated as constitutive biomarkers for pathogen resistance in trees (Witzell and Martín, 2008; McPherson et al., 2014; Conrad et al., 2017). Results of the canonical discriminant analysis showed that leaf phenolic profiles predicted well the phenotypic vitality of oaks, separating especially the low vitality trees from other groups. Nevertheless, the potential relation of the detected polyphenols (quercetin and kaempferol flavonoids) to tree vitality was not straightforward. While the levels tended to be highest in low vitality trees, the lowest levels were detected in medium vitality trees. This non-linearity may reflect the multiple roles of phenolics in plants. Given their potential role as constitutive biomarkers, it could have been expected to find the highest levels in the most vital trees. Yet, accumulation of phenolics may be induced as a protective response to oxidative stress, e.g., in connection to stress or wounding (Lattanzio et al., 2006; Eyles et al., 2010; Sherwood and Bonello, 2013). Possibly, the low vitality trees expressed stress at physiological level, which may have explained accumulation of phenolics in their leaves. The exact mechanism(s) behind the possible connection between phenolic metabolism and endophyte communities still remain to be studied.

## Conclusion

In conclusion, our results suggest that the xylem in trees with decreased vitality is a good substrate for endophytic diversity. The role of dead wood as a supporter for fungal diversity is well established (Bader et al., 1995; Lassauce et al., 2011), but our results suggest that even trees with lower vitality can be reservoirs or stepping stones for fungal endophytes in urban and peri-urban areas. This role is even more important in isolated trees and for fungi with limited dispersion capacity (Bolund and Hunhammar, 1999; Fahrig, 2003; Dearborn and Kark, 2009; Peay et al., 2012). For managers of urban and peri-urban areas, the challenge will be to provide a suitable habitat for fungal biodiversity specific to trees with different gradients of vitality and at the same time ensure public safety in parks and densely populated areas. More detailed studies focusing on endophytes network and substrate utilization would be needed to understand the ecological and managerial implications of the endophyte–host interactions on ecosystems.

## Data Availability Statements

The raw data supporting the conclusions of this manuscript will be made available by authors, without undue reservation, to any qualified researcher.

## Author Contributions

MA and JW designed the experiments and conducted the sampling. MA conducted the laboratory work. MA, JM, BA, and JW analyzed the data. MA wrote the first draft of the manuscript. JW wrote sections of the manuscript. MC, JM, and BA provided valuable comments on the manuscript. All authors contributed to manuscript revision, read and approved the submitted version.

## Conflict of Interest Statement

The authors declare that the research was conducted in the absence of any commercial or financial relationships that could be construed as a potential conflict of interest.

## Footnotes

1 https://blast.ncbi.nlm.nih.gov/

## Abbreviations

CF: colonization frequency
S^MT^: morphotype richness
